# The relationship of redox signaling with the risk for atherosclerosis

**DOI:** 10.3389/fphar.2024.1430293

**Published:** 2024-08-01

**Authors:** Sujuan Lei, Chen Liu, Tian-xiang Zheng, Wenguang Fu, Mei-zhou Huang

**Affiliations:** ^1^ Department of General Surgery (Hepatopancreatobiliary Surgery), The Affiliated Hospital, Southwest Medical University, Luzhou, China; ^2^ Metabolic Hepatobiliary and Pancreatic Diseases Key Laboratory of Luzhou City, Academician (Expert) Workstation of Sichuan Province, Department of General Surgery (Hepatopancreatobiliary Surgery), Chongqing, Sichuan, China

**Keywords:** atherosclerosis, redox signaling, antioxidants, vascular homeostasis, atherogenic risk factors

## Abstract

Oxidative balance plays a pivotal role in physiological homeostasis, and many diseases, particularly age-related conditions, are closely associated with oxidative imbalance. While the strategic role of oxidative regulation in various diseases is well-established, the specific involvement of oxidative stress in atherosclerosis remains elusive. Atherosclerosis is a chronic inflammatory disorder characterized by plaque formation within the arteries. Alterations in the oxidative status of vascular tissues are linked to the onset, progression, and outcome of atherosclerosis. This review examines the role of redox signaling in atherosclerosis, including its impact on risk factors such as dyslipidemia, hyperglycemia, inflammation, and unhealthy lifestyle, along with dysregulation, vascular homeostasis, immune system interaction, and therapeutic considerations. Understanding redox signal transduction and the regulation of redox signaling will offer valuable insights into the pathogenesis of atherosclerosis and guide the development of novel therapeutic strategies.

## 1 Introduction

Oxidative balance plays a crucial role in physiological homeostasis (Kattoor et al., 2017; Smit et al., 2020; Batty et al., 2022). The regulation of oxidative balance influences cellular processes, including DNA replication, mRNA transcription, and protein synthesis; cell proliferation, differentiation, and death; and cellular function execution (Silverman et al., 2016). Recently, fields such as oxidative biology and oxidative chemistry have expanded our understanding of oxidative stress in disease (Libby et al., 2002; Martin et al., 2013; Jain et al., 2015). A strong link has been established between oxidative imbalance and various diseases, particularly age-related conditions (Meydani, 2004; Mathew et al., 2012; Choghakhori et al., 2023; Nazari-Bonab et al., 2023).

Oxidative stasis becomes more fragile with age due to diminished antioxidant response and increased oxidant activity. Advancements in real-time detection technologies have highlighted the role of oxidative signaling in disease progression (Li and Pagano, 2017; Greaney et al., 2019; Zhang et al., 2020; Omar et al., 2022). Oxidative status not only reflects changes in the body’s physiological state but also triggers signal transduction for physiological or pathological outcomes (Kinkade et al., 2013; Altenhöfer et al., 2015; Chocry and Leloup, 2020; Feiteiro et al., 2022). While oxidative regulation in disease contexts is understood, there remains a need for unbiased recognition of the role of oxidative stress in both physiological and pathological conditions. Antioxidant therapies have emerged as common treatments for age-related diseases, yet a comprehensive understanding of oxidative stress in diverse environments is essential.

Previous reviews on oxidative stress and atherosclerosis have primarily focused on the role of oxidative stress in lipid peroxidation, inflammation, and the recruitment of immune cells to the lesion sites (Scatena et al., 2010; Förstermann et al., 2017; Paulo et al., 2020; Schlapbach et al., 2022). However, a comprehensive analysis of the distinct mechanisms through which redox signaling influences the progression and outcome of atherosclerosis, especially in terms of the interplay between oxidative stress and immune cells, remains underexplored. Our review aims to bridge this gap by providing insights into the impact of redox signaling on risk factors such as dyslipidemia, hyperglycemia, and unhealthy lifestyle, and its effects on vascular homeostasis, immune system interaction, and the choice of antioxidant/pro-oxidant therapeutic approaches. Understanding redox signaling dynamics and their role in atherosclerosis will offer novel insights and expand current therapeutic strategies.

## 2 The relationship of redox signaling with risk factors for atherosclerosis

### 2.1 Dyslipidemia and oxidation homeostasis in atherosclerosis

Dyslipidemia caused by lipid metabolism disorders is the primary incentive for atherosclerotic formation and development (Ohara et al., 1993; Rizzo et al., 2009; Tietge, 2014). The ability of dyslipidemia to induce vascular endothelial cell injury, abnormal thickening of vascular smooth muscle, and infiltration of inflammatory cells has been reviewed in detail (Table 1) (Niebauer et al., 2001; Rizzo et al., 2009; Tietge, 2014; Beverly and Budoff, 2020). Oxidative stress is not only an initiator but also an executor, which is implicated in the pathological process of dyslipidemia-induced atherosclerosis. Excessive lipid metabolites, especially low-density lipoprotein cholesterol (LDL-C), triglyceride (TG) and total cholesterol (TC), are abnormally modified by vascular endothelial cells (ECs), inflammatory cells and smooth muscle cells, leading to the generation of peroxide or superoxide products, including lipid peroxidation products and reactive oxygen species (Table 1) (Hjuler Nielsen et al., 2015; Summerhill et al., 2019; Sukhorukov et al., 2020; Ji et al., 2021).

**TABLE 1:** Summary of preclinical and clinical findings on dyslipidemia, hyperglycemia and oxidative stress in atherosclerosis.

Aspect	Preclinical findings	Clinical findings
Lipid Metabolism	Induces vascular endothelial cell injury, thickening of vascular smooth muscle, inflammatory cell infiltration	Positive association between oxidative status in plasma and lipoprotein levels in atherosclerosis
Oxidative Modification	Generation of peroxide or superoxide products	Disturbance in mitochondrial membrane fluidity and composition leading to ROS production and mtDNA damage
Cell Damage	Induces damage to ECs, smooth muscle cells, and immune cells	Evidence of oxidative stress linking AGEs, PKC activation, polyol pathway flux, and hexosamine pathway flux in atherosclerosis
AGEs and ROS	AGEs stimulate ROS generation or disturb redox balance	Clinical findings confirm AGEs role in causing vascular dysfunction

The oxidative modification of LDL is crucial in atherosclerosis. LDL undergoes oxidative changes in the arterial walls due to ROS and specific enzymes, forming oxidized LDL (oxLDL). OxLDL induces endothelial dysfunction, reduces nitric oxide (NO) production, and increases leukocyte adhesion. It also promotes adhesion molecule expression, recruiting monocytes that become macrophages and form foam cells, leading to fatty streaks. OxLDL triggers the release of pro-inflammatory cytokines, perpetuating inflammation, attracting immune cells, and promoting smooth muscle cell proliferation, resulting in plaque growth. As atherosclerosis progresses, unstable plaques form, leading to thrombosis and potential cardiovascular events like myocardial infarction or stroke (Xanthoulea et al., 2009; Förstermann et al., 2017). In summary, LDL oxidation and oxLDL formation drive endothelial dysfunction, inflammation, and foam cell formation, leading to plaque development and vascular complications. Understanding these processes is key to developing atherosclerosis treatments.

Lipoxygenases (LOXs) play critical roles in atherosclerosis by oxidizing polyunsaturated fatty acids to form bioactive lipid mediators. The two main isoforms, 5-lipoxygenase (5-LOX) and 12/15-lipoxygenase (12/15-LOX), have distinct functions. 5-LOX, primarily in leukocytes, converts arachidonic acid into leukotrienes, promoting inflammation, macrophage activation, foam cell formation, and vascular smooth muscle cell (VSMC) proliferation (Coffey et al., 2001a; Kühn et al., 2005). In contrast, 12/15-LOX, found in endothelial cells, VSMCs, macrophages, and platelets, oxidizes arachidonic acid to 12-HETE and 15-HETE, leading to LDL oxidation, foam cell formation, and endothelial dysfunction. These enzymes contribute to plaque instability and progression by driving inflammation and cellular changes within the arterial wall (Othman et al., 2013; Ibrahim et al., 2015). Targeting 5-LOX and 12/15-LOX offers potential therapeutic strategies to mitigate inflammation and atherosclerotic plaque development in cardiovascular disease.

**FIGURE 1:**
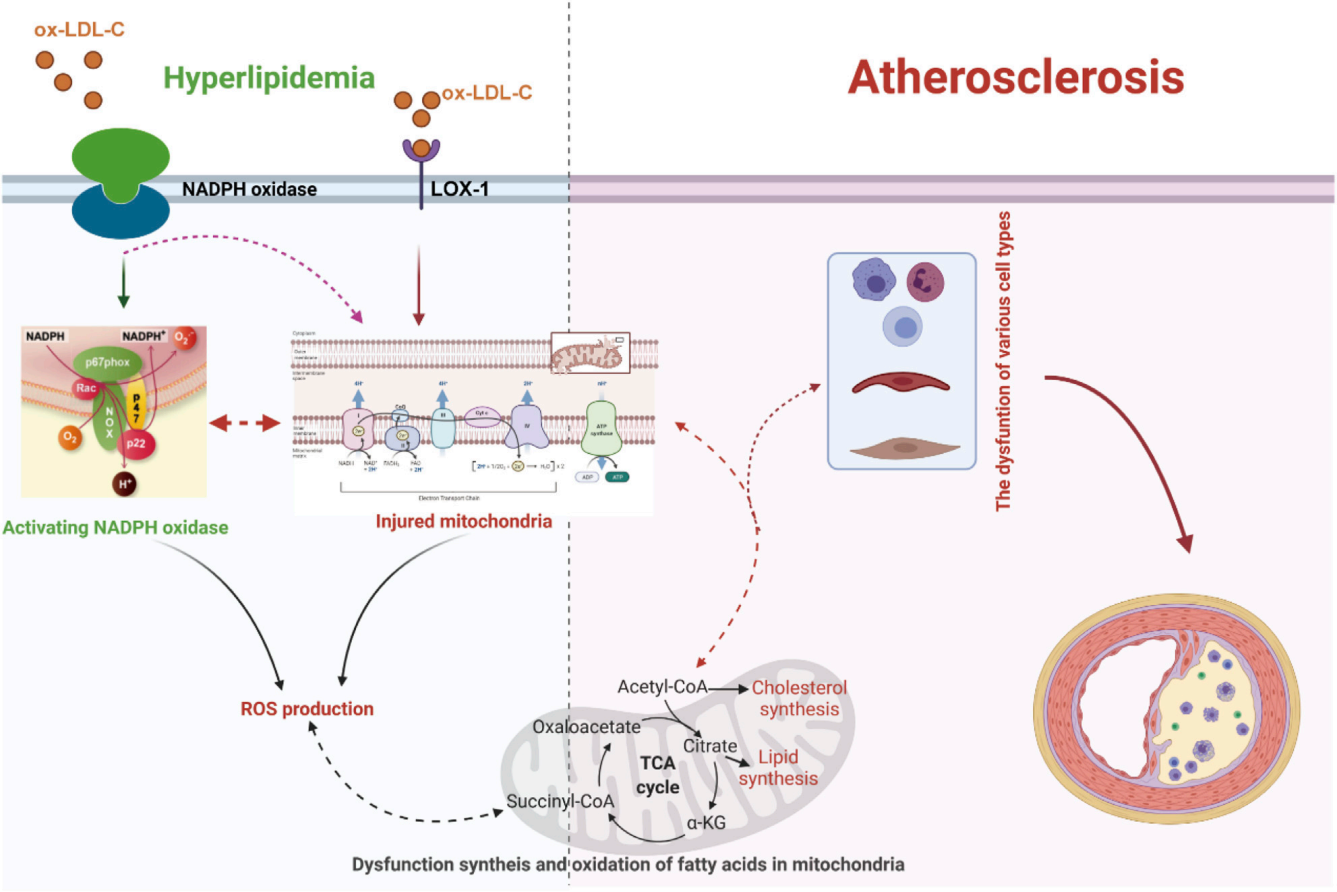
Dyslipidemia-enhanced ROS production via disruption of the mitochondrial respiratory chain and activation of NADPH oxidase can reinforce endothelial permeability to low-density lipoprotein cholesterol (LDL-C), facilitate LDL-C peroxidation, and facilitate differentiation of inflammatory cells, resulting in the release of proinflammatory factors and the expression of adhesion factors, which further boost the progression of dyslipidemia-induced atherosclerosis. LOX-1, lectin-like oxidized low-density lipoprotein receptor-1; ox-LDL-C, oxidative low-density lipoprotein cholesterol.

It has been verified that excessive lipid metabolites lead to an increase in the compensatory oxidation of mitochondrial fatty acids and an excess of acetyl-CoA, which leads to an increase in nicotinamide adenine dinucleotide (NADH) produced by the tricarboxylic acid cycle and an increase in the electron supply of mitochondria (Noctor et al., 2015; Ahotupa, 2017; Kastaniotis et al., 2017; Gordaliza-Alaguero et al., 2019) (Figure 1). Some high-energy electrons overflow from the chain, producing the reactive oxygen species (ROS). Excessive ROS production will damage the antioxidant defense system, leading to oxidative stress. Moreover, hypercholesterolemia enhances endothelial superoxide anion production by activating xanthine oxidase, causing vascular endothelial cell oxidative injury and then accelerating the occurrence and development of atherosclerosis (Ohara et al., 1993; Cai and Harrison, 2000; Miller et al., 2010; Shen, 2010; Konior et al., 2014). In addition, the accumulation of excessive free fatty acids (FFAs) in adipocytes leads to the activation of nicotinamide adenine dinucleotide phosphate (NADPH) oxidase and the production of excessive ROS (Han et al., 2012) (Figure 1). Clinical studies have also documented strong positive associations between the oxidative status in plasma and the lipoproteins level in atherosclerosis patients (Table 1) (Mahapatra et al., 1998; Dandana et al., 2011). Under conditions of hyperlipidemia, the fluidity and lipid composition of the mitochondrial membrane are also disturbed, which leads to the production of a large amount of ROS and impairs mitochondrial DNA (mtDNA). Injury to mtDNA exacerbates mitochondrial dysfunction and affects the synthesis and oxidation of fatty acids in mitochondria, which contributes to a further deterioration of dyslipidemia-induced atherosclerosis (Oliveira and Vercesi, 2020).

In brief, the alteration of oxidation homeostasis has been involved in the whole progression of dyslipidemia-induced atherosclerosis. The cooperation of dyslipidemia with oxidative stress expedites the onset and development of atherosclerosis.

### 2.2 Hyperglycemia and oxidative homeostasis in atherosclerosis

**FIGURE 2:**
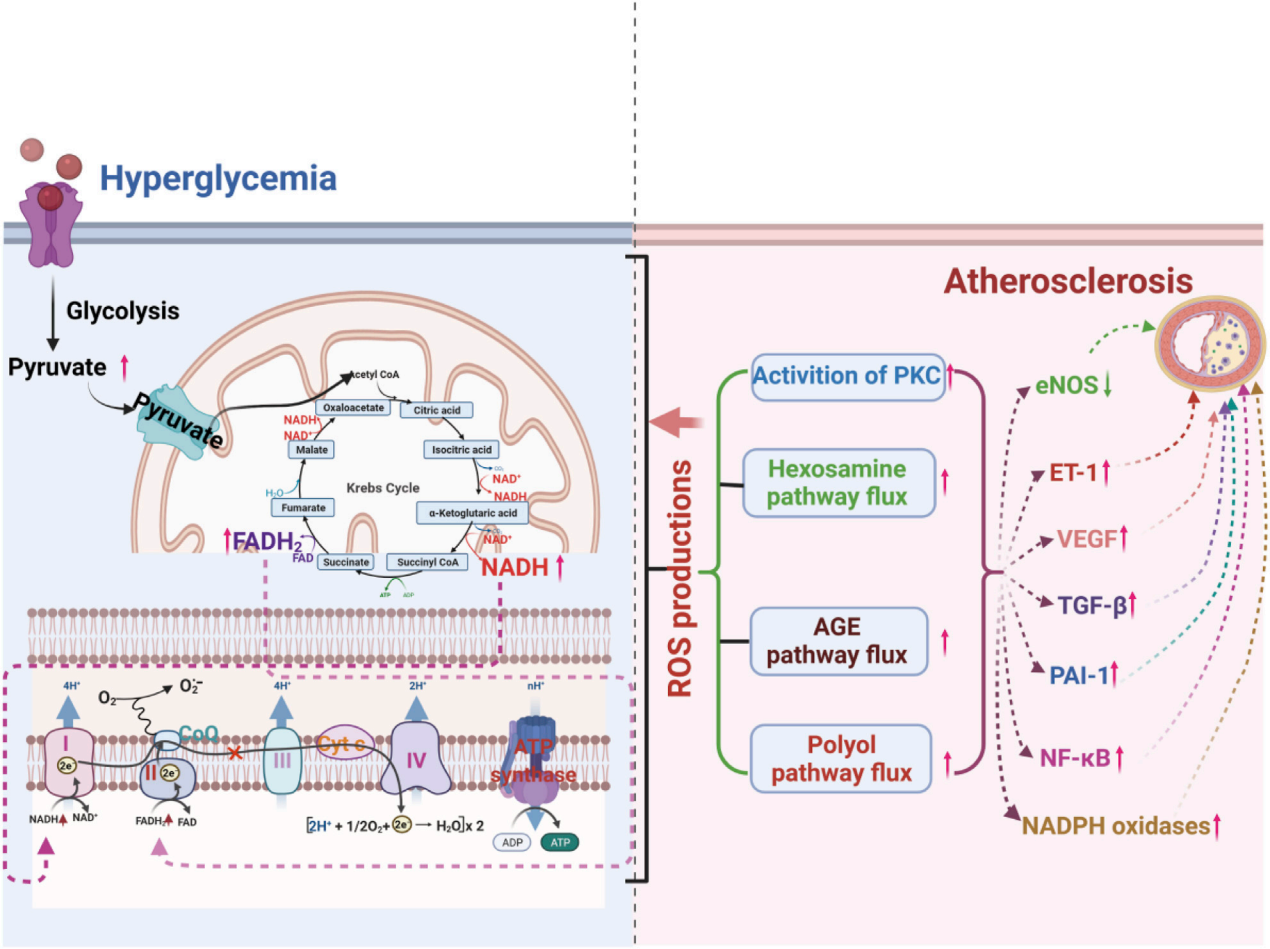
ROS production from hyperglycemia-injured mitochondria plays a crucial role in hyperglycemia-induced atherosclerosis by increasing the flux of the polyol pathway, the formation of advanced glycation end-products (AGEs), the activation of protein kinase C (PKC) isoforms, and the flux of the hexosamine pathway.

Hyperglycemia is an independent risk factor for atherosclerosis, but it also interacts with other risk factors. It is well known that hyperglycemia can induce damage to multiple cell types, including ECs, smooth muscle cells, and immune cells, and promote the occurrence and development of atherosclerosis by disturbing multiple signaling pathways (Ott et al., 2014; Bifulco et al., 2017; Wang S. et al., 2020; Nyambuya et al., 2020). How does the diverse pathogenesis of atherosclerosis all result from hyperglycemia? There is an increasing consensus that increased advanced glycation end-products (AGEs) formation, activation of protein kinase C (PKC) isoforms, increased polyol pathway flux, and increased hexosamine pathway flux play vital roles in hyperglycemia-induced atherosclerosis (Brownlee, 2001; Chait and Bornfeldt, 2009; Bornfeldt and Tabas, 2011; Yuan et al., 2019). A growing body of evidence suggests that oxidative stress may play a key linking hub in the four pathways mentioned above of hyperglycemia-induced atherosclerosis (Giacco and Brownlee, 2010; Tang et al., 2012; Papachristoforou et al., 2020) (Figure 2).

AGEs can stimulate the generation of ROS or disturb the redox balance, causing vascular dysfunction (Del Turco and Basta, 2012; Wang X. et al., 2020). It has been reported that in a hyperglycemic environment, increased AGEs can bind to AGE receptors, inducing receptor-mediated production of ROS and promoting the uncoupling of nitric oxide synthase, leading to redox imbalance (Barlovic et al., 2011; Yamagishi and Matsui, 2018). Hyperglycemia also accelerates intracellular AGE formation by ROS-dependent processes, causing damage to target cells. The inhibition of glyceraldehyde-3-phosphate dehydrogenase (GAPDH) by mitochondrial overproduction of ROS resulting from hyperglycemia increases triose phosphate levels and then promotes the generation of AGEs (Table 1) (Du et al., 2000; Del Turco and Basta, 2012; Sergi et al., 2021). Increasing AGEs can aggravate ROS formation (Du et al., 2000; Brownlee, 2001).

Activation of PKC mediated by hyperglycemia can affect the permeability of vascular ECs, systolic and diastolic balance of vascular smooth muscle, blood flow, and aggregation and invasion of inflammatory cells, causing vascular dysfunction (Koya and King, 1998; Geraldes et al., 2009; Volpe et al., 2018). High glucose may enhance ROS production through PKC-dependent activation of NOX or by directly activating NOX (Giacco and Brownlee, 2010). Excessive ROS production upregulates related genes and oxidatively modified related proteins, promotes the accumulation of monocytes into blood vessels to strengthen the local inflammatory response and alter the normal function of ECs, promoting the progression of atherosclerosis (Förstermann et al., 2017; Kattoor et al., 2017; Ochoa et al., 2018). Hyperglycemia can also disrupt the mitochondrial electron transport chain and lead to excess electron binding with O_2_ to O_2_ (Cardoso et al., 2013; Rehni et al., 2015). Mitochondrial superoxide overproduction may initiate the hyperglycemia-induced *de novo* synthesis of diacylglycerol or phosphatidylcholine hydrolysis that activates PKC (Nishikawa and Araki, 2007; Nishikawa et al., 2007). The reduction of superoxide overproduction by corresponding inhibitors can significantly alleviate the activation of PKC (Rolo and Palmeira, 2006; Wang et al., 2013).

Hyperglycemia-induced increases in polyol pathway flux strongly stimulate and promote endothelial dysfunction and vascular alterations (Kitada et al., 2010; Ramasamy and Goldberg, 2010). Oxidative stress plays a significant role in hyperglycemia-induced vascular injury by increasing the polyol pathway flux (Bonnefont-Rousselot, 2002). Hyperglycemia strengthens the affinity of aldose reductase for glucose, resulting in increased polyol pathway flux with the accompanying depletion of NADPH (Ramasamy and Goldberg, 2010). Decreased NADPH can obstruct the synthesis and recycling of antioxidants, including reduced glutathione (GSH) and glutathione oxidase, causing the accumulation of superoxide (Brownlee, 2001). Moreover, hyperglycemia-induced overproduction of superoxide can enhance polyol pathway flux by inhibiting GAPDH-induced accumulation of glycolytic metabolites (Gaskin et al., 2001; Yuan et al., 2019). Hyperglycemia-induced sorbitol accumulation is wholly prevented by thenoyltrifluoracetone (TTFA), uncoupling protein 1 (UCP-1) and manganese superoxide dismutase (Mn-SOD), indicating that mitochondrial superoxide overproduction stimulates aldose reductase activity (Nishikawa et al., 2000).

In short, redox signal molecules, including H_2_O_2_, NO, and oxyradicals, act as critical signal transmitters and executors in the pathogenesis of hyperglycemia-induced vascular dysfunction.

### 2.3 Inflammation and oxidation homeostasis in atherosclerosis

There is a consensus that atherosclerosis is a chronic inflammatory disorder (Geovanini and Libby, 2018; Raggi et al., 2018; Ruparelia and Choudhury, 2020). An in-depth study of oxidative homeostasis in blood vessels has shown that the alteration of oxidation homeostasis in the vascular system contributes to the occurrence and development of atherosclerosis, drawing more attention (Salazar, 2018; Marchio et al., 2019). Recent research has revealed that the invasion and agglomeration of various inflammatory factors in the vascular system promote the progression of atherosclerosis by disturbing oxidation homeostasis. Simultaneously, excessive ROS formation by dysfunctional mitochondria, activation of phagocytic and nonphagocytic NOX enzymes, NADPH oxidase mitochondria, and uncoupling eNOS are influential promotion factors for a proinflammatory phenotype since they can activate immune cells and enhance the expression of cytokines, chemokines, molecule adhesion and other proinflammatory mediators in the vascular endothelium, thus contributing to the development of atherosclerotic lesions (Salazar, 2018; Wolf and Ley, 2019).

**FIGURE 3:**
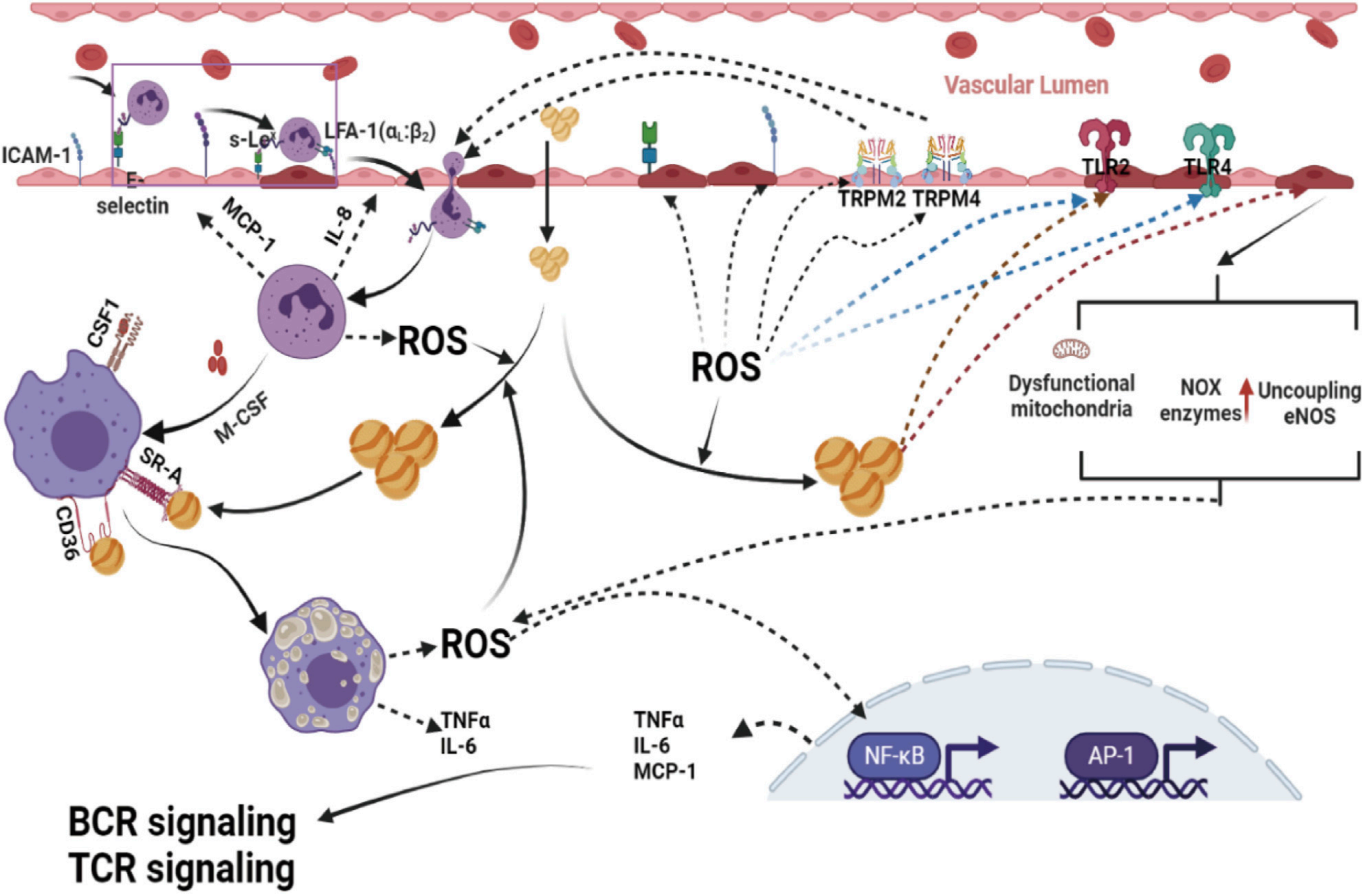
Excessive ROS formation by dysfunctional mitochondria, activation of phagocytic and nonphagocytic NOX enzymes, NADPH oxidase mitochondria, and uncoupling eNOS are powerful promotion factors for a proinflammatory phenotype, since they can activate immune cells and enhance the expression of cytokines, chemokines, molecule adhesion and other proinflammatory mediators in the vascular endothelium, and disrupt Ca^2+^ homeostasis in ECs by modulating transient receptor potential melastin 2 (TRPM 2) channels, which contribute to the migration of immune cells into subendothelial cells, leading to ECs thus contributing to the development of atherosclerotic lesions. ROS, reactive oxygen species; AP-1, activator protein 1; NF-κB, nuclear factor-kappa B; BCR, B-cell receptor; TCR signaling, T-cell receptor; TLR2, toll-like receptor 2; TLR4, toll-like receptor 4.

Excessive generation of ROS may induce endothelial oxidative injury, impair endothelial barrier function, enhance the expression of vascular adhesion factors, oxidize LDL-C in the blood, promote the adhesion and evasion of monocytes, and then induce the differentiation of monocytes into macrophages. It is becoming a widespread view that oxidative stress-mediated signaling pathways play a primary role in the regulation of inflammatory responses in the occurrence and development of atherosclerosis (Pignatelli et al., 2018; Khosravi et al., 2019) (Figure 3).

Redox-sensitive transcription factors, including activator protein 1 (AP-1) and nuclear factor-kappa B (NF-κB), are considered critical bridging factors between oxidative stress and inflammation in the occurrence and development of atherosclerosis (Reuter et al., 2010; El Assar et al., 2013). These redox-sensitive transcription factors activated by ROS can enhance the gene expression of proinflammatory factors [tumor necrosis factor-α (TNF-α), interleukin-1 (IL-1), and interleukin-6 (IL-6)], proinflammatory enzymes [inducible nitric oxide synthase (iNOS), cyclooxygenase-2 (COX-2)], adhesion molecules [intercellular adhesion molecular-1 (ICAM), vascular cell adhesion molecule (VCAM)], and antioxidant enzymes [Mn-SOD, NOQ-1 and glutathione peroxidase (Gpx)], one of the primary steps in the formation and development of lesions. In addition, there is an emerging opinion that the development of inflammatory reactions in atherosclerosis is a compensatory response for defense against oxidative stress-induced blood vessel injuries.

Recently, there has been an emerging view that transient receptor potential melastatin channels also play an essential role in oxidative stress-induced inflammation. Oxidative stress can disrupt Ca^2+^ homeostasis in ECs by modulating transient receptor potential melastatin 4 (TRPM 4) channels, which contribute to the migration of immune cells into subendothelial cells, leading to ECs (Sarmiento et al., 2015). Transient receptor potential melastatin 2 (TRPM 2), another member of the transient receptor potential melastatin family, is also involved in ROS-induced inflammatory reactions by regulating monocyte chemokine production (Yamamoto et al., 2008; Takahashi et al., 2011). Experimental, clinical, and population studies have demonstrated that increased antioxidant protection of injured blood vessels by enhanced expression of superoxide dismutase (SOD) and GPX significantly reduces the inflammatory reaction (Hadj Ahmed et al., 2018; Mayyas et al., 2018).

In summary, the causal relationship between oxidative stress and vascular inflammation in the occurrence and development of atherosclerosis is difficult to understand due to the numerous interplays between these two factors. Nevertheless, it is widely accepted that inflammation and oxidative stress are the primary drivers of the chronic inflammatory reaction that is characteristic of atherosclerosis.

### 2.4 Unhealthy lifestyle and oxidation homeostasis in atherosclerosis

Oxidative stress caused by smoking is generally considered a significant independent risk factor for atherosclerosis (Niemann et al., 2017). There are approximately 10^17^ oxygen-free radicals in each cigarette’s gas phase and tar. ROS are a primary component of cigarette smoke, which can settle in the bloodstream and lead to vascular oxidative injury by oxidative modification of macromolecules (Yang et al., 2017; Golbidi et al., 2020). Additionally, ROS present in cigarette smoke can reduce nitric oxide (NO) bioavailability by uncoupling eNOS and causing vascular dysfunction (Golbidi et al., 2020).

The ingredients in cigarette smoke, including nicotine and coal tar, can also induce and activate ROS-producing enzyme systems, such as NAD(P)H oxidase and xanthine oxidase, within the vessel wall (Niemann et al., 2017; Wu X. et al., 2018; Wang et al., 2019). In addition, the components of cigarette smoke can also lead to mitochondrial dysfunction and subsequently aggravate ROS generation, which is considered the most crucial trigger of atherosclerosis (Dikalov et al., 2019). Altogether, these findings suggest that oxidative stress plays a central role in accelerated cardiovascular aging in smokers.

Increased vascular oxidative stress is an essential mediator in ethanol-induced cardiovascular diseases (Wu et al., 2006; Liang et al., 2014). It has been documented that ethanol-induced hypertension and endothelial dysfunction are closely related to an increase in ROS generation (Carnevale and Nocella, 2012; Matyas et al., 2016). The overproduction of ROS induced by ethanol causes vascular oxidative stress; activates redox-sensitive signaling pathways, including lipid peroxidation and protein oxidation; and leads to elevations in intracellular calcium ([Ca^2+^]), reduced nitric oxide (NO) bioavailability, proinflammatory cytokine production, the mitogen-activated protein kinases activation, and ultimately vascular inflammation and endothelial dysfunction (Wu et al., 2006; Bau et al., 2007; Carnevale and Nocella, 2012).

Recent findings suggest that the increase in vascular ROS in subjects with alcoholism is associated with dysfunctional mitochondria, activated NOX and xanthine oxidase (XO), uncoupled e-NOS, and a dysregulated antioxidant system induced by ethanol (Ceron et al., 2014; Piano, 2017; Simplicio et al., 2017). Ethanol elicits a decrease in the activity of mitochondrial respiratory chain complexes due to alterations in mitochondrial ribosomes, which decreases electron transfer efficiency and leads to ROS formation (Hoek et al., 2002). In addition, ethanol can deplete the mitochondrial antioxidant system by reducing the activity of Mn-Superoxide dismutase (Mn-SOD), GPx, and catalase (Cat), which leads to the accumulation of overproduced ROS in mitochondria and exacerbates mitochondrial dysfunction (Ceron et al., 2014). Altogether, these alterations may promote oxidative injury to blood vessels, thus contributing to the progression of alcohol-induced cardiovascular diseases.

Sedentary lifestyle-induced oxidative stress causes endothelial dysfunction, arterial wall stiffening, and a quantifiable impairment of vascular function, which contribute to the development of cardiovascular diseases, including atherosclerosis and hypertension (Lessiani et al., 2016). There are various mechanisms for sedentary lifestyle-induced oxidative stress (Carter et al., 2017). Physical inactivity can decrease mitochondrial content and damage mitochondrial function, which causes excessive ROS generation and affects numerous physiological functions and disease states. Moreover, physical inactivity also enhances vascular NOX expression and activity, which results in enhanced vascular ROS production. In addition, physical inactivity can reduce blood flow and local shear stress, which will induce the production of ROS (Aseervatham et al., 2013).

In brief, unhealthy lifestyle-induced oxidative stress caused by various mechanisms, including impaired mitochondrial function, activated ROS-producing enzyme systems, eNOS uncoupling, and antioxidant system disruption, has been generally recognized as an independent risk factor for the occurrence and development of cardiovascular diseases.

## 3 Dysregulation of redox signaling and vascular homeostasis in atherosclerosis

### 3.1 Hemodynamics and redox signals in atherosclerosis

Established evidence indicates that hemodynamic characteristic forces play a vital role in sustaining vascular structure and function (Gimbrone and García-Cardeña, 2013; Inoue, 2014). Physiological hemodynamic forces, including steady laminar shear stresses and pulsatile shear, can be transduced into biochemical signals to trigger multiple downstream signaling pathways and maintain vascular homeostasis (Ando and Yamamoto, 2022). However, pathologic forces, such as oscillatory shear stresses, may alter cellular mechanotransduction and lead to the occurrence and development of cardiovascular diseases (Fan et al., 2019; Ando and Yamamoto, 2022). Atherosclerosis is prone to occur at the branches of blood vessels in which multidirectional blood flow can easily lead to fluctuations in hemodynamics (Gimbrone and García-Cardeña, 2013; Heo et al., 2014; Inoue, 2014). Moreover, ample evidence also confirms that the alteration of blood hemodynamics is an initial incentive for atherosclerosis.

Various signaling pathways are implicated in regulating redox status in response to altered blood hemodynamics (Chatterjee and Fisher, 2014). The orchestration of ROS-producing and antioxidant systems has been recognized as a central role in the interaction of blood hemodynamics and vascular oxidative homeostasis. Under physiological conditions, steady laminar stresses can cause a transient induction of NOX activity that sustains the lower level of ROS to act as a messenger in downstream signal transduction and maintain vascular oxidative homeostasis. However, low or high laminar stresses and oscillating shear stresses, lead to sustained NOX activity and O_2_^−^ production.

Oscillatory shear stress, acute shear stress, and low shear stress can rupture the cellular balance among XO, xanthine oxidoreductase (XOR), xanthine dehydrogenase (XDH), and NOX, causing excessive ROS production generation (Raaz et al., 2014).

In addition, blood hemodynamics can regulate the vascular redox status by manipulating mitochondrial homeostasis (Scheitlin et al., 2014; Wu L.-H. et al., 2018). Emerging studies have revealed that shear stress can reinforce tricarboxylic acid cycle activity under physical conditions and strengthen electron flow to electronic transport chains by transiently increasing intercellular calcium, which can induce ROS formation (Scheitlin et al., 2016; Yamamoto et al., 2018). Although laminar shear stress induces transient increases in ROS, mitochondria remain healthy because shear stress can also increase NO production by activating eNOS and upregulate antioxidant genes, which contributes to scavenging increased ROS and retaining membrane polarity. However, acute shear stress induces dysfunction of mitochondrial complexes I and III, causing the accumulation of excessive ROS in mitochondria (Takabe et al., 2011; Scheitlin et al., 2014). In addition, emerging studies have shown that oscillatory flow can induce ROS overproduction in mitochondria via the NOX-JNK (c-Jun NH2-terminal kinase) signaling pathway (Takabe et al., 2011). In addition, low shear stresses also promote mitochondrial ROS generation and induce vascular injury by the loss of ten-eleven translocation-mediated upregulation of the expression and activity of mitochondrial respiratory complex II subunit succinate dehydrogenase B (SDHB) (Scheitlin et al., 2014; Wu L.-H. et al., 2018).

Recent reports have verified that blood hemodynamics regulate not only ROS generation but also affect the antioxidant system. In particular, the activity of several antioxidant enzymes, including heme oxygenase 1 (OH-1), SOD, thioredoxin reductase, and glutathione peroxidase, is closely related to blood hemodynamics (Inoue et al., 1996; Takeshita et al., 2000; Jones et al., 2007). Steady shear stresses can enhance the activity of SOD, while oscillatory shear stresses reduce the activity of glutathione peroxidase and catalase (Jones et al., 2007).

Overall, these combined findings explain how steady and disrupted flow produce distinguishing effects on ROS generation and antioxidant enzymes, overturning the balance toward an antioxidative environment in the former condition and a prooxidative state in the latter.

### 3.2 Cross-regulation of oxidative signals and vascular endothelial cells in atherosclerosis

Redox homeostasis in ECs is finely regulated by coordinated ROS generation and a perfect antioxidant system that has been well-reviewed in previous reports (Maron and Michel, 2012; Panieri and Santoro, 2015; Aldosari et al., 2018). Since many vascular risk factors can lead to an increase in ROS, ROS was also initially considered a risk factor for damage to vascular endothelial cell homeostasis. Interestingly, compelling evidence indicates that a suitable amount of ROS is a prerequisite for vascular endothelial homeostasis (Fukai and Ushio-Fukai, 2020; Obradovic et al., 2020). The dual roles of ROS in vascular tissues depend on the spatiotemporal features and content of ROS produced in the cell. Under physiological conditions, because adequate ROS exhibit targeting, specificity and, compartmentalization, they play a crucial role in endothelial cell signal transduction to regulate endothelial structure and function, including endothelial cell polarization, proliferation, migration, apoptosis and senescence permeability (Panieri and Santoro, 2015). In addition, under pathological conditions, excessive ROS leads to endothelial cell dysfunction through either enzyme-mediated or enzyme-independent mechanisms (Wolin, 2009).

In brief, it has become clear that redox signaling can mediate atherogenic risk factor-induced endothelial injury signal transduction, causing the development of atherosclerosis (Gimbrone and García-Cardeña, 2016). This process is achieved by activating various redox-sensitive kinases (Madamanchi and Runge, 2013), such as protein tyrosine phosphatases (PTPs), controlling phosphorylation-dephosphorylation reactions; modifying ion channel properties, such as SR/ER Ca^2+^-ATPase (SERCA) pumps; and altering transcription factor activity, including NF-κB, AP-1, hypoxia-inducible factor-1 (HIF-1), p53 and E26 transformation-specific or E-twenty-six (ETS) (Lee et al., 2021).

### 3.3 Oxidative signals stimulate the diversity of smooth muscle cells in atherosclerosis

With atherogenic risk factor stimuli, VSMCs can undergo phenotypic switching and subsequently migrate toward the intima, synthesize excess extracellular matrix, and release inflammatory cytokines by either redox signaling-mediated or redox signaling-independent mechanisms (Badran et al., 2020). It has long been established that redox signaling is implicated in the progression of atherogenic risk factor-mediated dysfunction of VSMCs.

The atherogenic risk factor-impaired VSMC redox balance characterized by excessive ROS directly or indirectly affects VSMC phenotypic switching, proliferation, migration, and apoptosis, which play essential roles in mediating, integrating, and executing atherogenic signal transduction (San Martín and Griendling, 2010; Gomez and Owens, 2012; Grootaert et al., 2018). The redox balance of VSMCs is finely tuned by a diversified ROS production system and sophisticated antioxidant system (Durgin and Straub, 2018).

In VSMCs, the ROS production system consists of the mitochondrial respiratory chain, xanthine oxidase, lipoxygenases, uncoupling nitric oxide synthases, cytochrome p450 monooxygenase, hemoxygenases and NADPH oxidases (Clempus and Griendling, 2006; Vara and Pula, 2014). Moreover, these ROS sources, such as hemoxygenases and NADPH oxidases, have tissue and subcellular localization specificity to sustain the specificity and compartmentalization of the oxidation state of VSMCs (Vara and Pula, 2014). NADPH oxidase-derived ROS modulate the signaling pathways of VSMCs in a paracrine, autocrine, or even intracrine manner (Nowak et al., 2017). However, excess ROS is responsible for converting vascular smooth muscle cells from physiological to pathological roles. The close relationship of oxidative stress with the heterogeneity of VSMCs, a critical step in the occurrence and development of atherosclerosis, establishes a connection between ROS production and VSMC proliferation, hypertrophy, migration, and secretion of extracellular matrix (San Martín and Griendling, 2010; Badran et al., 2020).

ROS can, directly and indirectly, regulate lamellipodia formation, actin cytoskeleton remodeling, focal adhesion turnover, and cell body contraction through alterations of intracellular signaling pathways during specific migration phases (Weber et al., 2004). For instance, several migratory stimuli, including platelet-derived growth factor (PDGF) with the corresponding receptor (PDGF-R), lead to the generation of ROS over minutes to hours (Mbong and Anand-Srivastava, 2012; Salabei et al., 2013). These ROS can induce activation of the long form of slingshot-1 (SSH1L) by oxidizing an inhibitory partner protein of slingshot phosphatases (SSH), 14-3-3, which is responsible for cofilin activation and focal adhesion turnover (Kim et al., 2009). In VSMCs, ROS can affect intracellular Ca^2+^ release and calcium influx through oxidation-modified calcium channels and calcium ion regulatory proteins, which play a vital role in regulating cell body contraction (Trebak et al., 2010; Zhao et al., 2020). Overproduction of ROS can directly oxidize β-actin, and this oxidative modification has been implicated in polymerization, lamellipodia formation, and actin cytoskeleton remodeling in VSMCs (San Martín and Griendling, 2010; Wilson et al., 2016; Xu et al., 2017; Badran et al., 2020). Moreover, ROS directly or indirectly affect VSMC proliferation and apoptosis depending on the spatiotemporal content and duration of the ROS produced (San Martín and Griendling, 2010; Badran et al., 2020).

These findings indicate that several atherogenic stimuli can induce positive redox feedback in VSMC transformation from physiology to pathology. Not only do these ROS mediate atherogenic stimuli signal transduction in VSMCs, but they also participate in the execution of the pathological function of VSMCs.

### 3.4 Crosstalk between oxidative signals and perivascular adipose tissue in atherosclerosis

Since the inside-outside signaling paradigm was recognized in the development of atherosclerosis, the changes in vascular endothelial function were thought to be the critical regulator to trigger atherosclerosis (Ross, 1999; Chen et al., 2020). Perivascular adipose tissue (PVAT) has long been assessed to be nothing more than vessels supporting connective tissue and protecting the blood vessels from the stretching of surrounding tissue (Szasz and Webb, 2012). Recently, studies have demonstrated that PVAT could also influence the atherosclerotic process, and the inside-outside signaling paradigm of atherosclerosis development was re-evaluated (Iacobellis et al., 2008; Verhagen and Visseren, 2011; Bartuskova et al., 2022). The critical role of PVAT in the development of atherosclerosis has been gradually emphasized and reviewed (Ichiki, 2010; Verhagen and Visseren, 2011; Chang et al., 2013; Nosalski and Guzik, 2017). PVAT could influence endothelial function, regulate contraction, proliferation, and migration of VSMC, and induce an inflammatory response to vascular injury by meticulous biological regulation pathway (Nóbrega et al., 2019; Zhu et al., 2019; Bartuskova et al., 2022; Dos Reis Costa et al., 2022; Man et al., 2022). Crosstalk between oxidative signals and PVAT in atherosclerosis is drawing more and more people’s attention.

Like other vascular components, including endothelium and VSMC, PVAT also possesses abundant resources of ROS, including mitochondria, NADPH oxidase, and uncoupled eNOS, which is necessary for PVAT to participate in vascular physiological and pathological processes (Gao et al., 2006; Costa et al., 2016; Xia et al., 2016). It is well documented that under physiological conditions, homeostatic ROS, namely, eustress, plays a vital role as signal transduction molecules in PVAT, maintaining vascular health (Gao et al., 2006). Pieces of evidence from other studies have suggested that H_2_O_2_ from PVAT could regulate vasodilation-independent intervention of vascular endothelium (Costa et al., 2016). Physiological ROS from PVAT is also indispensable for VSMC contraction, proliferation, and migration (Gao et al., 2006; Costa et al., 2016). The antiatherosclerotic role of PVAT-derived adiponectin was associated with ROS signaling transduction in vascular tissue. PVAT could upregulate adiponectin-gene expression in response to the alteration in the vascular redox hemostasis (Margaritis et al., 2013). In turn, PVAT-derived adiponectin negatively regulates vascular superoxide formation by enhancing eNOS phosphorylation and tetrahydrobiopterin bioavailability and reducing eNOS uncoupling in vascular cells (Margaritis et al., 2013). It has been clarified that the interaction of mitoNEET with NADPH oxidase plays an essential role in maintaining vascular thermogenesis and redox balance of PVAT by affecting thermogenic genes, including UCP-1, Pgc1α, Cox8b, and Elovl3 and proinflammatory factors such as monocyte chemoattractant protein 1 (MCP-1), IL-6, and TNF-α (Didion, 2017; Xiong et al., 2017). Although homeostatic ROS acting as a signaling transductor is undoubtedly crucial to vascular homeostasis, it is becoming increasingly distinct that there is considerably more to the biological effect of PVAT-derived ROS.

An altered redox status in the PVAT in response to atherogenic factors, including obesity, hyperlipidemia, aging, and other stimuli, might signal to the surrounding vascular tissue and incline to the development of atherosclerosis (Xia et al., 2016; da Costa et al., 2017; Bailey-Downs et al., 2013). It has been well documented that atherogenic factors such as obesity could upregulate the expression of mineralocorticoid receptors in PVAT, and the upregulation of mineralocorticoid receptors enhances the generation of PVAT-derived ROS by activating NADPH oxidase, enhancing eNOS uncoupling and inducing mitochondrial dysfunction (Hashikabe et al., 2006; Leopold et al., 2007; Calhoun and Sharma, 2010). Moreover, slimming might reduce the mineralocorticoid receptors in PVAT and restore PVAT redox status and vascular physiological function (Crissey et al., 2014). These findings suggested that PVAT oxidative stress is an essential mediator in obese-induced vascular injury and atherosclerosis. Recently, it is well known that the PVAT, due to their sophisticated cell members, and various properties and functions, possesses various underdetermined mechanisms, in addition to releasing various factors by affecting endocrine and paracrine mechanisms to regulate vascular function in response to atherogenic factors (Fernández-Alfonso et al., 2017; Huang Cao et al., 2017). The PVAT oxidative stress appears as a pathophysiological mechanism underlying vascular dysfunctions in response to atherogenic factors (Victorio and Davel, 2020). However, the mechanisms underlying PVAT oxidative stress in atherogenic factors are needed to elucidate it.

Considering the importance of PVAT-derived ROS in regulating vascular homeostasis, targeted regulation of the oxidative state of PVAT has gradually become a new focus of antioxidant therapy for atherosclerosis (Nacci et al., 2016; Schinzari et al., 2017; Man et al., 2020). In *in vitro* studies and animal work, some antioxidants, including mitoNEET ligands and sirtuin-1 activators, were used to treat vascular dysfunction disease by regulating the redox status of PVAT and achieved a positive effect (Minor et al., 2011; Geldenhuys et al., 2014; Yoo et al., 2015). However, the detailed mechanisms of antioxidant treatment in PVAT are needed to elucidate, and further development is needed in targeted antioxidants for PVAT. Moreover, it is critical to delineate the receptors on ECs, VSMCs, and immune cells that receive the PVAT-derived redox signals.

## 4 The interaction of redox signaling with immune systems in atherosclerosis

### 4.1 Innate immune systems and redox signals in atherosclerosis

Compelling studies have clarified that innate immune systems play a vital role in the pathogenesis of atherosclerosis, including the induction of inflammation and the formation of fatty streaks and plaques (Mendel et al., 2015; Schaftenaar et al., 2016; Pirillo et al., 2018). Extracellular and intracellular signals, such as lipopolysaccharides and oxidative low-density lipoprotein cholesterol (ox-LDL-C), induce the activation and differentiation of diverse cells from the innate immune system. These activated immune cells subsequently secrete proinflammatory or anti-inflammatory factors to facilitate the progression of atherosclerosis (Mendel et al., 2015; Pirillo et al., 2018; Herrero-Fernandez et al., 2019). ROS boosts the interaction of the innate immune system and atherosclerosis with different roles as initiators, mediators, or effectors (Miller et al., 2011; Chakrabarti and Visweswariah, 2020; Sun et al., 2020).

Innate immunity plays a crucial role in the occurrence and development of atherosclerosis, starting from the prime events of the expression of adhesion molecules in ECs, chemokine release, monocyte recruitment, and macrophage activation to intricate cellular interactions in mature atherosclerotic plaques (Kobayashi et al., 2005; Riksen and Stienstra, 2018; Moroni et al., 2019; Pirro and Mannarino, 2019; van Tuijl et al., 2019). In atherosclerosis, many studies have posited that innate immune cells can be divided into professional innate immune cells, such as mast cells, monocytes, neutrophils, macrophages, and dendritic cells, and conditional innate immune cells, including ECs and smooth muscle cells (Hu et al., 2019; van Tuijl et al., 2019; Hofbauer et al., 2022). Recent studies recognized EC and smooth muscle cells as conditional innate immune cells that share most features of professional innate immune cells in response to extracellular environmental changes (Hu et al., 2019; Shao et al., 2020). These innate immune cells are the source of ROS products and the target of ROS products. NOX and mitochondria are two primary sources of increased ROS production in innate immune cells, which play critical roles in regulating innate immunity (Benmoussa et al., 2018; Breda et al., 2019; Al-Shehri et al., 2020; Trevelin et al., 2020).

In atherosclerosis, professional and conditional innate immune cells rapidly recognize and respond to atherogenic signals derived from injured self-cells or metabolic stresses through damage-associated molecular patterns or metabolic sensors (MSs) (Griffiths et al., 2017; Riksen and Stienstra, 2018; van Tuijl et al., 2019). Activated innate immune cells release proinflammatory or anti-inflammatory cytokines, chemokines, C-reactive protein, and ROS production that initiate signaling cascades and promote further recruitment and activation of other immune cells. It has also been clarified that redox signaling plays a critical role in these biological processes (Lauridsen, 2019; Sun et al., 2020).

Compelling studies have certified that the ROS produced by innate immune cells, including macrophages, neutrophils, monocytes, dendritic cells, and mast cells, can signal atherogenic factor stimuli, mediate cell-cell interactions and promote immune cell activation and differentiation (Jaganjac et al., 2016; Zeng et al., 2019; Chakrabarti and Visweswariah, 2020; Li et al., 2020). For instance, excessive LDL-C can induce macrophages to produce ROS products, and these produced ROS, in turn, change macrophage subsets in response to alterations in the level of LDL-C (Tavakoli and Asmis, 2012). Likewise, neutrophils are potent sources of ROS, affecting the function of neutrophils, activated macrophages, and subsequent foam cell formation (Tavakoli and Asmis, 2012). Two signals are considered to represent the mechanism by which ROS drives the activation, differentiation, and intercommunication of innate immune cells: signal 1, oxidative modification of critical enzymes and transcription factors by oxidation-sensitive thiol groups; signal 2, glutathionylation of associated functional molecules by regulation of the level of GSH (Butturini et al., 2020; Mullen et al., 2020). Pioneering studies have also suggested that during proinflammatory immune responses, most immune cells switch toward enhanced glycolysis and the pentose phosphate pathway to facilitate the activation and differentiation status increasing cytosolic and mitochondrial ROS production plays a vital role in metabolic reprogramming (Kelly and O’Neill, 2015; Groh et al., 2018). These phenomena indicate that redox regulation may occur at several steps in the immune signaling pathways.

### 4.2 Adaptive immune systems and redox signals in atherosclerosis

Following an innate immune response manifested as activation of professional or conditional innate immune cells in the vessel wall, antigen-specific adaptive immune responses are evoked and mediated by T and B-cells. Redox signaling, acting as a messenger, plays a vital role in bridging the innate immune and adaptive immune responses in the occurrence and development of arteriosclerosis (Table2) (Sun et al., 2020). Compelling studies have confirmed that redox signaling is involved in the engagement of the T/B-cell receptor (TCR/BCR) by antigen peptides on the major histocompatibility complex (MHC), cytokine stimulation, and metabolite-associated danger signal (MADS) recognition by MSs (Gringhuis et al., 2002; Verweij and Gringhuis, 2002; Sun et al., 2020). It is well known that antigen peptides on major MHC, cytokine stimulation and MADS recognition by metabolic sensors are significant pathways that link innate and adaptive immune responses (Sun et al., 2020).

**TABLE 2:** Summary of preclinical and clinical findings on vascular homeostasis, immune system interaction and oxidative stress in atherosclerosis.

Aspect	Preclinical findings	Clinical findings
Endothelial Function	Oxidative stress reduces NO bioavailability and increases superoxide production	Reduced NO bioavailability and increased oxidative stress linked to endothelial dysfunction in patients
Peroxynitrite Formation	Imbalance between NO and superoxide leads to peroxynitrite formation	Interventions enhancing NO bioavailability show promise in clinical settings
Immune Cell Modulation	Oxidative stress modulates activity of macrophages and T-cell	Elevated markers of oxidative stress and inflammation observed in patients
Inflammatory Response	Oxidative stress enhances recruitment of inflammatory cells to lesions	Therapeutic approaches targeting oxidative stress reduce inflammation and improve outcomes

**FIGURE 4:**
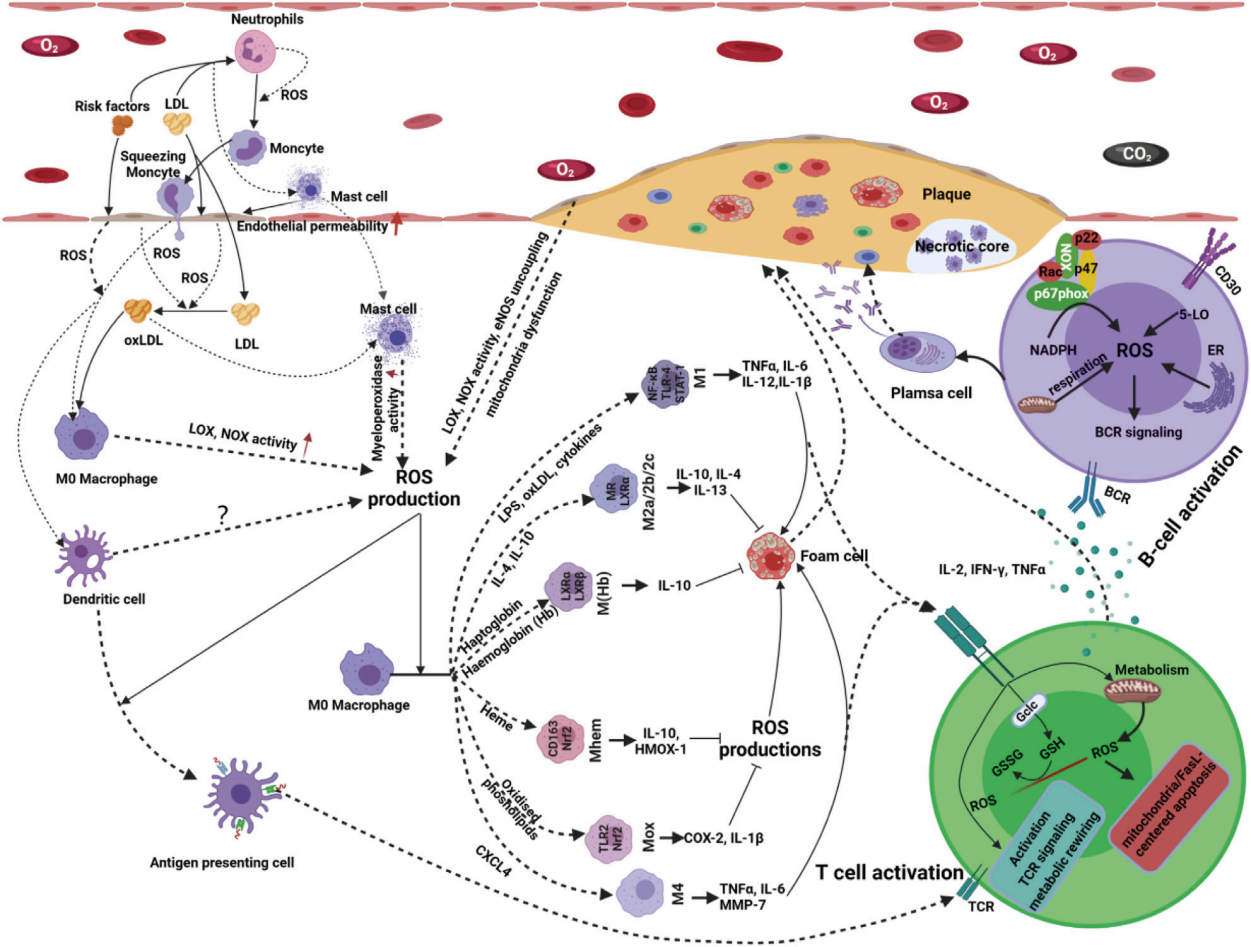
Innate immune cells serve as both the source and target of ROS products. Activated innate immune cells release proinflammatory or anti-inflammatory cytokines, chemokines, C-reactive protein and ROS production that initiate signaling cascades and promote further recruitment and activation of other immune cells. ROS produced by innate immune cells, including macrophages, neutrophils, monocytes, dendritic cells and mast cells, can signal atherogenic factor stimuli, mediate cell-cell interactions and promote immune cell activation and differentiation. Following an innate immune response manifested as activation of professional or conditional innate immune cells in the vessel wall, antigen-specific adaptive immune responses are evoked and mediated by T and B-cells. Redox signaling, acting as a messenger, plays a vital role in bridging the innate immune and adaptive immune responses in the occurrence and development of atherosclerosis. With the stimulation of atherogenic factors, T-cells are activated, accompanied by ROS production ROS production originating from T-cells has been implicated in T-cell biology, such as T-cell activation, proliferation, differentiation and apoptosis. Atherogenic factor-induced antigen stimulation enhances the production of ROS in B-cells via several alternative routes; thus, increases in ROS affect B-cell maturation, activation, proliferation, apoptosis and BCR signaling, resulting in even more ROS production.

Antigen presentation is a complex biological process involving antigen-presenting cells (APCs) and adaptive immune cells. ROS production plays a crucial role in the antigen presentation process in atherosclerosis (Figure 4). An imbalance in ROS production can significantly reduce the antigen uptake and presentation ability of dendritic cells (DCs) (Rutault et al., 1999; Martner et al., 2011; Kotsias et al., 2013). In contrast, moderate ROS production can promote the antigen-presenting ability of various APCs (Gong and Chen, 2003). Moreover, many APCs, including professional APCs (DCs, macrophages and B-cells) and nonprofessional APCs (ECs, VSMCs and other nucleated cells), are not only regulatory targets but also sources of ROS production (Matsue et al., 2003; Yan and Banerjee, 2010; Kotsias et al., 2013). Therefore, the fine regulation of ROS production shows broad prospects in immunotherapy for atherosclerosis.

Antigen presentation signals facilitate the activation and differentiation of resting B and T-cells upon antigen or mitogen stimulation. Extensive studies have shown that ROS production plays a crucial role in T/B-cell activation and TCR/BCR signaling (Barr et al., 2015; Lee et al., 2021).

With the stimulation of atherogenic factors, T-cells are activated, accompanied by ROS production (Franchina et al., 2018; Yarosz and Chang, 2018; Sánchez-Villanueva et al., 2019). It has been indicated that 5-lipoxygenase, NOX-2, dual oxidase-1 (DUOX-1), and mitochondria are important sources of ROS in T-cells in response to antigen presentation signals (Previte et al., 2017; Desdín-Micó et al., 2018; Yarosz and Chang, 2018). ROS production originating from T-cells has been implicated in T-cell biology, such as activation, proliferation, differentiation, and apoptosis (Belikov et al., 2015).

It has been reported that H_2_O_2_ originating from DUOX-1 can strengthen proximal TCR signaling, NOX-2-derived ROS can drive the differentiation of T-cells from the Th17 to the Th2 phenotype, and mitochondrial ROS production is indispensable for activation-induced IL-2 and IL-4 expression in resting and preactivated human T-cells (Kaminski et al., 2010; Kwon et al., 2010; Gill and Levine, 2013). In addition to T-cell activation, proliferation, and differentiation, ROS can induce T-cell apoptosis in response to microenvironmental changes via the extrinsic and intrinsic apoptotic pathways. Mitochondrial ROS and NOX-2-derived ROS trigger the expression of Fas ligand (FasL) and mitochondrial dysfunction, causing T-cell apoptosis (Williams and Kwon, 2004; Ueno et al., 2014; Deng et al., 2020). Moreover, since ROS can act as intercellular messengers, T-cell activation may also be mediated by ROS derived from surrounding cells (Yarosz and Chang, 2018). Overall, under physiological conditions, redox homeostasis is sustained by meticulous pro-oxidant and antioxidant systems, both in the vascular microenvironment and intracellular compartments, thus allowing normal T-cell responses to guarantee vascular physiological function. However, this delicate redox homeostasis is disturbed by the occurrence and development of atherosclerosis. Further exploration of the spatiotemporal dynamics and regulation of ROS in T-cells’ activation, proliferation, differentiation, and apoptosis and their microenvironment is essential for developing more effective treatments targeting atherosclerosis.

B-cells, another adaptive immune cell type, play a crucial role in the pathobiology of atherosclerosis (Tsiantoulas et al., 2014; Ma et al., 2021). B-cells have been detected in atherosclerotic plaques in humans and mice. Recent evidence has shown that ROS acts as secondary messengers to bridge the signal transduction of atherogenic factors, B-cell biology and atherosclerosis (Zhang et al., 2019; Ma et al., 2021). ROS increases rapidly in activated B-cells by atherogenic factor-induced antigen stimulation (Tsubata, 2020). There are multiple sources of ROS in B-cells in response to different stimuli, such as NOXs, mitochondria, 5-lipoxygenase (5-LO), and oxidative protein folding. Moreover, these sources of ROS are local and timely for sustaining the spatiotemporal transduction of redox signaling. For instance, early production of ROS in response to BCR stimulation originates from NOXs; at later stages of BCR stimulation, mitochondria are likely a significant source of ROS. During B-cell differentiation into plasma cells, the endoplasmic reticulum is an essential contributor to ROS production by oxidative protein folding (Figure 4). In atherosclerosis, the production of ROS and B-cell biology are reciprocal causations and functionally connected in a positive feedback loop (Vené et al., 2010; Bertolotti et al., 2012). Atherogenic factor-induced antigen stimulation enhances the production of ROS in B-cells via several alternative routes; thus, increases in ROS affect B-cell maturation, activation, proliferation, apoptosis and BCR signaling, resulting in even more ROS production (Vené et al., 2010; Bertolotti et al., 2012). Owing to the timeliness and concentration dependence of ROS-induced signaling in response to complex atherogenic factors, little is known about how ROS-induced signaling precisely regulates B-cell subset formation and B-cell metabolism in atherosclerosis. Therefore, gaps that need to be filled persist in this new area.

## 5 The choice of anti-/pro-oxidation in the treatment of atherosclerosis

To date, compelling studies have shown that the regulation of redox signaling is a desirable target for the management of atherosclerosis (Hu et al., 2000; Miller et al., 2011; Fullerton et al., 2013). Among various oxidation regulators, antioxidants are widely used in treating atherosclerosis as promising targeted regulatory molecules. The effect of all kinds of antioxidants on atherosclerosis has been well explored and reviewed (Table 3) (Mangge et al., 2014; Toledo-Ibelles and Mas-Oliva, 2018). Overall, to date, antioxidants targeted for the management of atherosclerosis can be divided into two categories: enzymatic antioxidants represented by SOD, Cat, GPx, thioredoxin reductase (TrxR), and nonenzymatic antioxidants, including endogenous nonenzymatic antioxidants represented by GSH, uric acid, bilirubin, coenzyme Q (CoQ)/CoQH2) and lipoic acid; and exogenous nonenzymatic antioxidants represented by α-tocopherol (vitamin E), ascorbic acid (vitamin C), B vitamins, carotenoids, and polyphenols, in addition to natural extracts that have also been used as antioxidants for the treatment of atherosclerosis (McCleery et al., 2018; Soto et al., 2020). Besides, both nitric oxide (NO) and nitro-fatty acids (NFAs) exhibit antioxidant and anti-inflammatory properties that counteract oxidative stress and inflammation, preventing LDL oxidation and atherosclerosis progression. NO directly scavenges reactive oxygen species (ROS), reducing oxidative stress and preventing the oxidative modification of LDL, thus reducing oxLDL formation. NFAs, with their strong electrophilic properties, neutralize ROS, preventing LDL oxidation and reducing pro-atherogenic oxLDL. NO, through its vasodilatory, antioxidant, and anti-aggregatory properties, and NFAs, through their antioxidant and anti-inflammatory actions, protect against atherosclerosis. Understanding these mechanisms offers potential therapeutic strategies to prevent or treat atherosclerosis by enhancing NO bioavailability and leveraging the benefits of NFAs. However, there is not a consensus regarding the beneficial effects of antioxidant therapies. In *in vitro* studies, animal work, and cross-sectional human studies, antioxidant treatment for atherosclerosis achieved a positive effect. Clinical interventional trials and epidemiologic cohort studies for the antioxidant treatment of atherosclerosis have not replicated these positive findings; moreover, in several clinical interventional trials, antioxidant treatment has shown adverse effects in managing atherosclerosis. No antioxidants are used to treat and prevent atherosclerosis in the clinic (Cherubini et al., 2005; Toledo-Ibelles and Mas-Oliva, 2018). Therefore, some researchers have advocated the termination of antioxidant treatment for atherosclerosis.

**TABLE 3:** Impact of pharmacological interventions on atherosclerosis.

Pharmacological intervention	Mechanism of action	Preclinical findings	Clinical findings
Statins (Kattoor et al., 2017; Smit et al., 2020; Batty et al., 2022)	Inhibition of HMG-CoA reductase	Reduce oxidative stress and inflammation, stabilize plaques, decrease LDL oxidation	Significant reduction in cardiovascular events, improved endothelial function, reduced inflammation (Libby et al., 2002; Martin et al., 2013; Silverman et al., 2016)
Antioxidants (e.g., Vitamin E, Vitamin C) (Mathew et al., 2012; Jain et al., 2015; Choghakhori et al., 2023)	Scavenging of free radicals	Some studies show reduced oxidative stress, while others show no significant effect	Mixed results in clinical trials; some studies show reduced oxidative markers, others show no clinical benefit (Meydani, 2004; Omar et al., 2022; Nazari-Bonab et al., 2023)
NADPH Oxidase Inhibitors (Li and Pagano, 2017; Greaney et al., 2019; Zhang et al., 2020)	Inhibition of NADPH oxidase	Reduction in ROS production, improved endothelial function, reduced plaque formation	Limited clinical data; early trials show promise in reducing oxidative stress markers (Kinkade et al., 2013; Altenhöfer et al., 2015; Chocry and Leloup, 2020)
NO Donors (Förstermann et al., 2017; Paulo et al., 2020; Feiteiro et al., 2022)	Enhancement of NO bioavailability	Improved endothelial function, reduced leukocyte adhesion, decreased plaque formation	Clinical trials show improved vascular function and reduced blood pressure (Scatena et al., 2010; Bondonno et al., 2012; Schlapbach et al., 2022)
Polyphenols (e.g., Resveratrol) (Nani et al., 2021; Parsamanesh et al., 2021; Gál et al., 2023)	Antioxidant and anti-inflammatory effects	Reduced oxidative stress, improved lipid profile, anti-inflammatory effects	Some clinical trials show improved endothelial function and reduced inflammatory markers (Kishimoto et al., 2013; Arinno et al., 2021; Moretti et al., 2024)
5-LOX Inhibitors (de Gaetano et al., 2003; Kobe et al., 2014; Gilbert et al., 2020)	Inhibition of leukotriene production	Decreased inflammation, reduced macrophage activation, decreased foam cell formation	Early clinical trials show potential in reducing inflammatory markers and improving lipid profiles (Becker et al., 2004; Hatmi et al., 2006; Saraf et al., 2022)
12/15-LOX Inhibitors (Coffey et al., 2001b; Conrad et al., 2010; Rai et al., 2014)	Inhibition of lipid peroxidation	Reduced LDL oxidation, decreased foam cell formation, reduced plaque stability	Limited clinical data; ongoing trials are investigating efficacy and safety (Belkner et al., 2005; Armstrong et al., 2016; Cakir-Aktas et al., 2023)

The failure of interventional and epidemiologic cohort studies to identify more positive results must be clarified. A careful reassessment of the evidence is warranted to understand the contradictory results. First, the classical oxidative stress hypothesis for atherosclerosis should be revisited. The classical oxidative stress hypothesis for atherosclerosis recognizes that an imbalance in the oxidation and antioxidant systems results in the occurrence and development of atherosclerosis. Extensive studies have found that free radical production plays an initial role as a cellular signaling messenger and that antioxidants exert regulatory, but not protective, functions in atherosclerosis (Siekmeier et al., 2007). Many researchers have proposed that the classical oxidative stress hypothesis in atherosclerosis should be replaced with the redox biology paradigm (Poznyak et al., 2020; Wang and Kang, 2020). It is necessary to understand the role of oxidative stress in standard processes and metabolic pathways. Although several methodologies have been used to study how redox reactions influence atherosclerosis carefully, the standardization of many of these methods still needs to be unified. In most interventional and epidemiologic cohort studies, antioxidants have been used as protective agents against free radicals, and their regulatory role in the oxidation state of diverse biological systems has been neglected. Interventions targeted to recover the endogenous antioxidant capacity and cellular stress response rather than exogenous antioxidants can reverse oxidative stress - halting a vicious cycle of inflammation and leading to atherosclerosis (Gale et al., 2001; Kishimoto et al., 2016). Antioxidants are targeted and specific as regulators of oxidative signals, and all antioxidants have the same function. Therefore, it is a prerequisite for ensuring the reliable results of clinical intervention experiments to choose appropriate antioxidants based on the characteristics of changes in the oxidation signal in atherosclerosis.

Second, since the body is an extensive oxidation system, the concentration and activity of specific antioxidants are easily blunted, preventing the critical active concentration of antioxidants at binding sites involved in the development of atherosclerosis. Developing a new antioxidant delivery pathway is essential to ensure the reliability and equity of antioxidants in clinical intervention studies in atherosclerosis.

Third, in clinical interventional studies, the diversity of enrolled subjects causes different responses to the same antioxidant (Willcox et al., 2008; Jain et al., 2015). Therefore, in clinical interventional studies, pharmacogenetic issues should be considered (Malekmohammad et al., 2019). When the regulatory mechanism of the differences in the effector function of antioxidants based on genetic profiles is better understood, many of the paradoxical findings may become apparent. Overall, the paradoxical results in epidemiologic studies and interventional trials should not impede the explosion of antioxidant therapies for atherosclerosis but rather facilitate the development of new antioxidant therapeutic schemes. Revisiting the results of epidemiologic studies and interventional trials will be beneficial to clarify further the role of antioxidants in the prevention and treatment of atherosclerosis. However, more extensive studies are required.

Additionally, the administration of antioxidants preventing and treating atherosclerosis can be accompanied by hormesis. Hormesis is a biphasic dose-response phenomenon where cells or organisms exposed to low doses of a chemical agent or condition produce stimulatory or adaptive beneficial effects, while higher doses produce inhibitory or toxic effects. The response to such a low dose of stress is considered to be an adaptive compensatory process or an adaptive stress response following an initial disruption of homeostasis, enhancing the organism’s ability to resist more severe stress (Calabrese et al., 2007; Mattson, 2008; Powers et al., 2020). Considering that the causes for the above are related to differences in antioxidant dosage and differences between individual redox status and atherosclerotic status, it is urgent to standardize and rationalize the relevant application of antioxidants.

The close relationship of redox status imbalance with the atherosclerotic status of patients, along with the evidence for the potential antioxidant atheroprotective role of several molecules, inspired us to explore redox regulator molecules for the prevention and treatment of atherosclerosis.

Although redox signal transduction plays a vital role in the occurrence and development of atherosclerosis, antioxidants related only to free radical scavengers might be unsuitable. Several antioxidant molecules can achieve their activity by affecting the expression pattern of proteomics and miRNAs that transform the cellular pattern into an antioxidant metabolism pattern (Salvayre et al., 2016). With the development of proteomics, metabolomics, transcriptomics, and multi-omics, changes in the metabolism of cells induced by a specific antioxidant treatment have been extensively elaborated. The cooperation of new methodologies with new concepts for antioxidant functions could significantly promote the application of antioxidants in the treatment of atherosclerosis and broaden the way to discover new antioxidants. For instance, synthetic active ingredients from natural extracts could not exert the antioxidant effects of natural extracts in several clinical intervention trials (Salvayre et al., 2016). Therefore, many researchers have proposed that antioxidant treatment for atherosclerosis is invalid (Willcox et al., 2008). However, the concept that natural extracts containing a synergistic active molecule can exert antioxidant effects in favor of undetected synergistic active molecules has drawn the attention of many researchers to revisit the aforementioned clinical intervention trials (Singh, 2019). The concept brands a new direction to explore potential antioxidant therapies for atherosclerosis, not as single molecules but as a synergistically protective natural mixture.

## 6 Summary and outlook

A distinctive feature is that the changes in steady oxidation in vascular tissue are almost synchronized with the atherogenic risk factor stimulus. Redox signaling can clearly mediate the signal transduction of hemodynamic disturbance, endothelial injury, VSMC dysfunction, and immune system imbalance induced by atherogenic risk factors by activating various redox-sensitive kinases, such as PTPs; controlling phosphorylation-dephosphorylation reactions; modifying ion channel properties, such as SERCA pumps; and altering transcription factor activity, including NF-κB, AP-1, HIF-1, p53, and ETS. Moreover, not only do these redox signalings mediate atherogenic stimuli signal transduction in VSMCs, but they also participate in the execution of the pathological function of hemodynamics, vascular ECs, VSMCs, and immune cells. Probing redox signal transduction and comprehending the net regulation of redox signaling is beneficial for clarifying the pathogenesis of atherosclerosis and developing new therapeutic strategies for atherosclerosis. However, persistent gaps need to be filled to achieve the aforementioned perfect expectation.

First, targeted quantitative real-time monitoring technology of the oxidation status in the body should be further developed to clarify the mechanism underlying the timeliness and concentration dependence of ROS-induced signaling in response to complex atherogenic factors. Second, further exploration of the spatiotemporal dynamics and regulation of ROS in the activation, proliferation, differentiation, and apoptosis of T-cells and their microenvironment is essential for the development of more effective treatments against atherosclerosis. In addition, owing to the timeliness and concentration dependence of ROS-induced signaling in response to complex atherogenic factors, little is known about how ROS-induced signaling precisely regulates B-cell subset formation and B-cell metabolism in atherosclerosis. Therefore, gaps remain that require filling in this new area. Third, finding an efficient way to target antioxidants to specific intracellular organelles, such as lysosomes and mitochondria, or to study subtle changes that might occur in the epigenetic control of gene expression secondary to antioxidant therapy could be considered two interesting approaches. We believe a comprehensive analysis of this new knowledge and its relationship with the presence of plasma antioxidants and their reducing capacity will undoubtedly open new avenues for understanding and developing novel therapeutic pathways to fight against atherosclerosis and other degenerative diseases.
